# CyberKnife Radiosurgery for Spinal Leptomeningeal Metastases Secondary to Esthesioneuroblastoma: A Clinical Case Report

**DOI:** 10.7759/cureus.39791

**Published:** 2023-05-31

**Authors:** Aroosa Zamarud, Ulas Yener, Rahman Sayed, Steven D. Chang, Antonio Meola

**Affiliations:** 1 Department of Neurosurgery, Stanford University School of Medicine, Stanford, USA; 2 Department of Neurosurgery, Montefiore Medical Center, Albert Einstein College of Medicine, Bronx, USA

**Keywords:** stereotactic radiosurgery (cyberknife®), leptomeningeal spread, leptomeningeal dissemination, leptomeningeal metas, cyberknife, stereotactic radiosurgery, olfactory neuroblastoma, esthesioneuroblastoma

## Abstract

Esthesioneuroblastoma (ENB), also known as olfactory neuroblastoma, is a rare malignant tumor of neuroectodermal origin that arises from the olfactory epithelium. We present a case of ENB metastasizing through the leptomeningeal route to the spinal dura, which was treated with CyberKnife (CK) stereotactic radiosurgery (SRS), and aim to assess the safety and effectiveness of SRS in such cases. To the best of our knowledge, this is the first case report in the literature that discusses ENB spinal leptomeningeal metastases treated with CK radiosurgery. We retrospectively review the clinical and radiological outcomes in a 70-year-old female with ENB metastasis to the spine. Progression-free survival (PFS), overall survival (OS), and local tumor control (LTC) are investigated.

In our patient, ENB had been diagnosed at the age of 58 years and spinal metastases had been first noted at the age of 65 years. A total of six spinal lesions received CK SRS. Lesions were present at the level of C1, C2, C3, C6-C7, T5, and T10-11. The median target volume was 0.72 cc (range: 0.32-2.54). A median marginal dose of 24 Gy was delivered to the tumors with a median of three fractions to a median isodose line of 80% (range: 78-81). LTC at the 24-month follow-up was 100%. PFS and OS were 27 months and 40 months, respectively. No adverse radiation effects were reported. Even though the treated spinal lesions remained stable, the number of new metastatic lesions had increased with progressive osseous and dural metastatic lesions within the cervical, thoracic, and lumbar spine at the last follow-up. SRS provides relatively good LTC for patients with ENB metastasizing to the spine, with no radiation-induced adverse events.

## Introduction

Esthesioneuroblastoma (ENB) or olfactory neuroblastoma is a rare and malignant cancer of the sinonasal tract. This area is in the roof of the nasal cavity, which separates the nasal cavity from the brain. Due to its rare and complex nature, varying opinions exist regarding the etiology, optimal staging system, and treatment modality of ENB. The estimated incidence of ENB is four per 10 million individuals [1] and it accounts for approximately 5% of all sinonasal tumors and 0.3% of all upper aerodigestive tract malignancies. Although it may occur at any age, it is predominantly reported in young adults [2]. The mean age of presentation is 40-70 years. Men and women are equally affected. It was first reported by Berger and Luc in 1924, after which over 1000 cases have been reported in the literature [2,3]. Its incidence seems to have increased over the last decade. Patients with ENB generally present with nasal obstruction, epistaxis, discharge, anosmia, and unilateral polyp. Facial swelling, pain, anesthesia, trismus, proptosis, extraocular movement paralysis, and blindness are the other manifestations [4].

ENB is considered curable thanks to the recent advances in both surgical and radiation techniques that have led to improved survival and local control, with a reported disease-free survival of 80.4% at eight years in a cohort of 35 patients [4]. Craniofacial resection combined with radiotherapy is reported as the gold standard of care [5]. It is a locally aggressive tumor and can also metastasize to distant locations both via hematogenous and lymphatic routes [1]. Systemic metastases occur in 10-30% of patients [6-8]. Metastases to the neck and cervical lymph node are by far the most commonly reported.

Leptomeningeal dissemination of ENB is extremely rare. The literature contains only a handful of case reports on this topic [9-12]. All the reported cases were treated with either radiotherapy and/or chemotherapy. We report a case of ENB with leptomeningeal metastases to the cervical, thoracic, and lumbar vertebral spaces, which was treated with CyberKnife (CK) stereotactic radiosurgery (SRS). We retrospectively review the clinical and radiological outcomes in the patient. Patient demographic data, clinical characteristics, treatment methods, observation period, and survival are investigated.

## Case presentation

**Figure 1:**
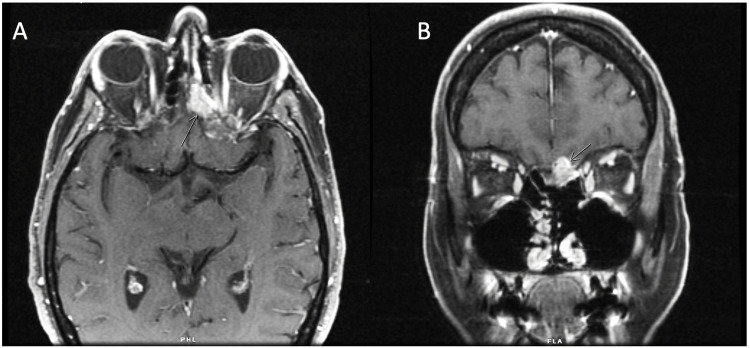
Esthesioneuroblastoma with intracranial extension Axial T1 (A) and cortical T1 (B) with contrast showing a contrast-enhancing mass centering on the left cribriform plate extending to the left ethmoid sinus with intracranial extension

The patient was a 70-year-old female, who had ENB metastasizing to the spinal cord dura. At the age of 58 years, she had presented to the Ear, Nose, and Throat (ENT) clinic with a history of terrible tooth pain for a few years. After extensive workup with her dentist, she had been referred to ENT for evaluation of her sinuses. She had undergone left-sided functional endoscopic sinus surgery for chronic sinusitis, nasal polyposis, and chronic rhinosinusitis, as well as the placement of a frontal stent. Pathology reported a left sinus, low-grade neuroendocrine tumor. After a year, she had presented again with headaches and retro-orbital pain. MRI scan had revealed a contrast-enhancing mass centering on the left cribriform plate extending to the left ethmoid sinus with intracranial extension (Figure 1). The mass had been noted to extend along the medial floor of the left anterior cranial fossa. Destruction of the cribriform plate had been appreciated. A biopsy had confirmed a neuroectodermal tumor. Additional presenting symptoms had been as follows: significant pain behind her left eye and in the back of her head, intermittent numbness of her lower left lip, pain in her neck and back, as well as dry eyes and mouth at night. Her tumor stage had been T4 N0.

**Figure 2:**
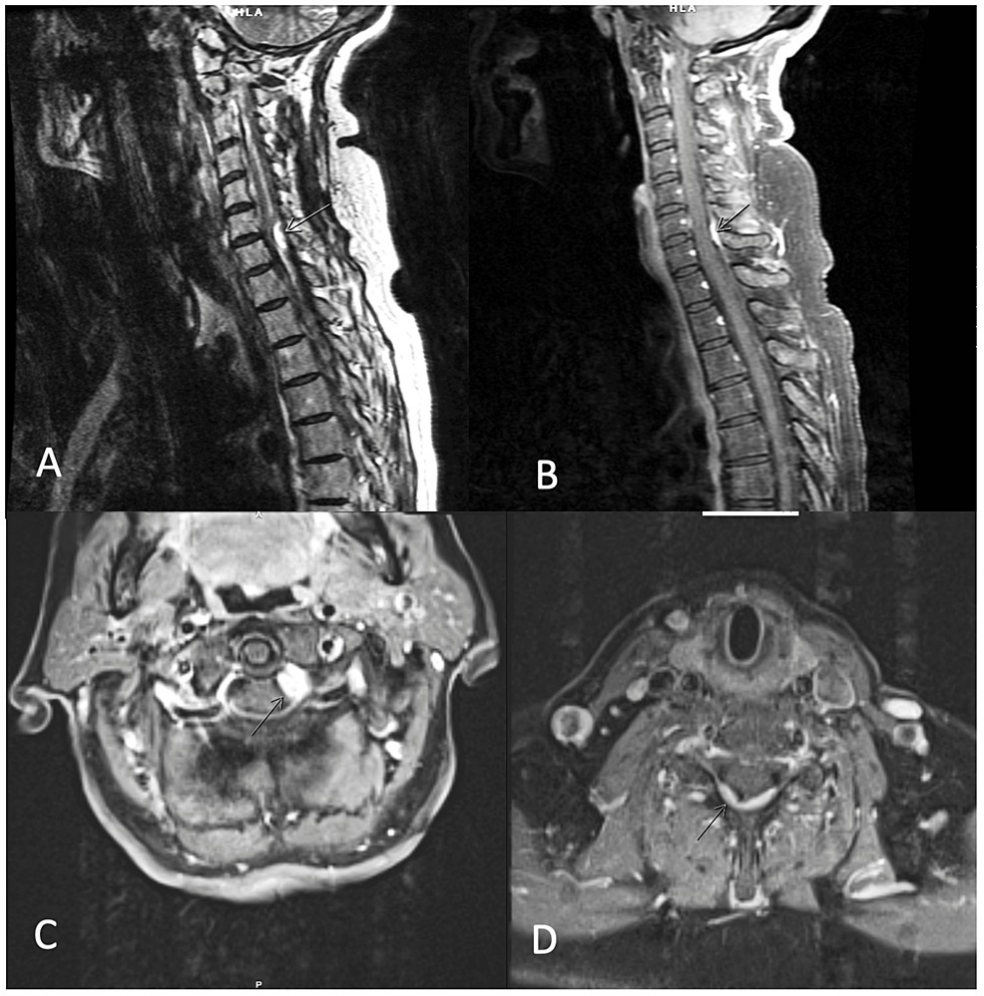
Spinal metastases secondary to esthesioneuroblastoma MRI scan demonstrating dural enhancement at T5 level; A*:* T2 scan with contrast, B*:* T1 scan with contrast MRI scan demonstrating dural enhancement at C1 level; C*:* T1 scan with contrast and C6-C7 level, D*:* T1 scan with contrast MRI: magnetic resonance imaging

She had undergone craniofacial resection, followed by postoperative radiation therapy for her primary lesion (50 Gy in 28 fractions). This had been followed by multiple recurrences and resections (endoscopic sinus procedures) (Table 1). Spinal dural metastases had been first diagnosed at the age of 65 years, when an MRI complete spine had demonstrated dural metastases to C1 and mild C6-C7, T5, and T9-T10 dural enhancement (Figure 2). She had been asymptomatic at the time, and her Eastern Cooperative Oncology Group (ECOG) score had been 0. The C1 lesion had been treated with SRS. A marginal dose of 24 Gy had been delivered in three fractions to an isodose line of 80%. The lesion had remained stable for two years, after which an increase in the C1 lesion, as well as C6-C7 and T9-T11 enhancement, had been noted. The C1, C2, C3, C6-C7, and T10-T11 dural lesions had again been treated with SRS, with a median marginal dose of 24 Gy, in three fractions to a median isodose line of 80%, and a median conformality index of 1.79. Around the same time, multiple new metastatic dural lesions had been noted in the cervical and thoracic spine. Her neurological symptoms, however, had been stable. At the six-month follow-up, an MRI scan showed that all the lesions except for C1 had been stable. C1, however, had demonstrated a slight increase in size. At nine-and 12-month follow-ups, all the lesions had been stable, and no new lesions had been noted. MRI at the 18-month follow-up, however, had shown that even though the previously treated lesions had been stable, there had appeared many new dural metastatic lesions at the cervical, thoracic (T9, T10-11), and lumbar spine.

**Table 1:** Clinical case details CK SRS: CyberKnife stereotactic radiosurgery; C: cervical; T: thoracic

Date, age in years	Diagnosis	Location	Treatment
May 2010, 58	Esthesioneuroblastoma	Left ethmoid cavity	Resection
June 2011, 59	Recurrent esthesioneuroblastoma extending into the brain parenchyma	Left cribriform plate	Surgical resection (endoscopically from below and through craniotomy from above) and postoperative radiation
September 2012, 60	Recurrent esthesioneuroblastoma	Left paranasal sinus	Surgical endoscopic resection
January 2014, 62	Recurrent esthesioneuroblastoma	Left neck	Left neck selective dissection
April 2016, 64	Progressive disease (anterior falx increased thickening)	Anterior sagittal falx	CK SRS (24 Gy in 3 fractions) to a volume of 1.1 cc, 0.3 cc, 0.2 cc, and 0.2 cc
July 2016, 64	Increase in left retropharyngeal node	Left retropharyngeal node	Left retropharyngeal lymph node dissection
July 2017, 65	Spinal dural metastases	Left C1 dural lesion, C6–C7, and T5 dural enhancement	CK SRS (24 Gy in 3 fractions) to a volume of 1.33 cc
October 2017, 65	Dural esthesioneuroblastoma recurrence	Left frontal and left temporal lobe	CK SRS (24 Gy in 1 fraction) to a volume of 0.615 cc and 1.8 cc
September 2018, 66	Esthesioneuroblastoma progression	Left frontotemporal, sagittal sinus, left temporal, and right temporal	CK SRS (24 Gy in 3 fractions) to a volume of 4.49 cc and 1.57 cc; CK SRS (21 Gy in 1 fraction) to a volume of 0.45 cc and 1.11 cc
April 2019, 67	Spinal dural metastases	C1, C2, C3, T10–T11 dural metastases	CK SRS (24 Gy in 3 fractions) to a volume of 0.63 cc, 0.72 cc, and 0.32 cc
April 2019, 67	Esthesioneuroblastoma cranial metastases	Superior sagittal and left frontal	CK SRS (24 Gy in 3 fractions) to a volume of 9.40 cc and 0.17 cc
April 2019, 67	Esthesioneuroblastoma progression	C6 and C7	CK SRS (24 Gy in 3 fractions) to a volume of 2.54 cc
December 2020, 68	Progressive spinal dural lesions	T9, T10–T11, throughout the thoracic and cervical spine	

Results

**Figure 3:**
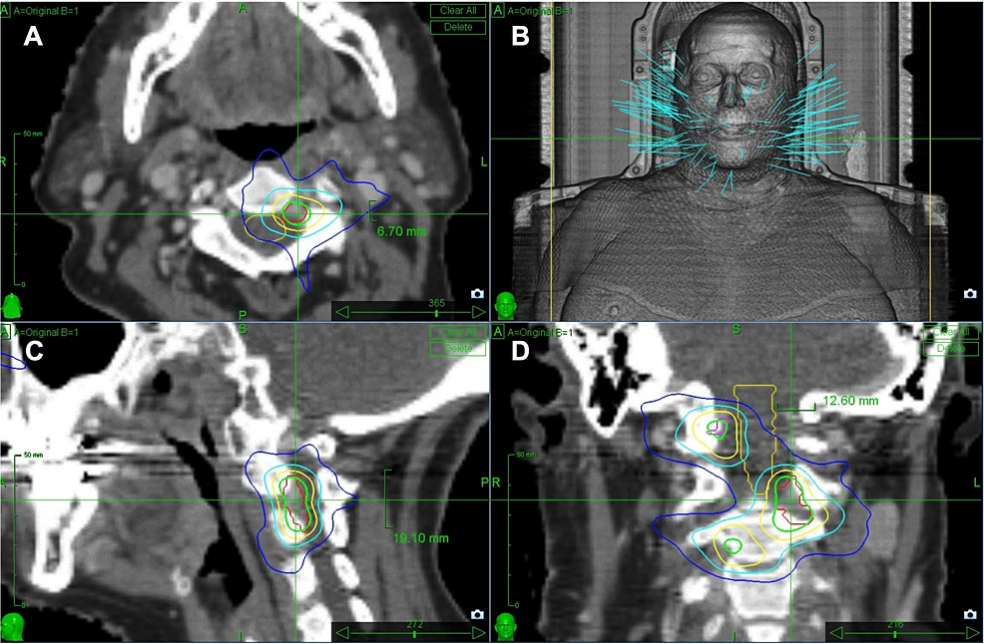
CyberKnife radiosurgery plan targeting left C2 metastases, using 3 fractions with a marginal dose of 24 Gy (D max 30 Gy), at 81% isodose line to a target volume of 0.72 cc Axial (A), sagittal (B), and coronal (D) sections

**Figure 4:**
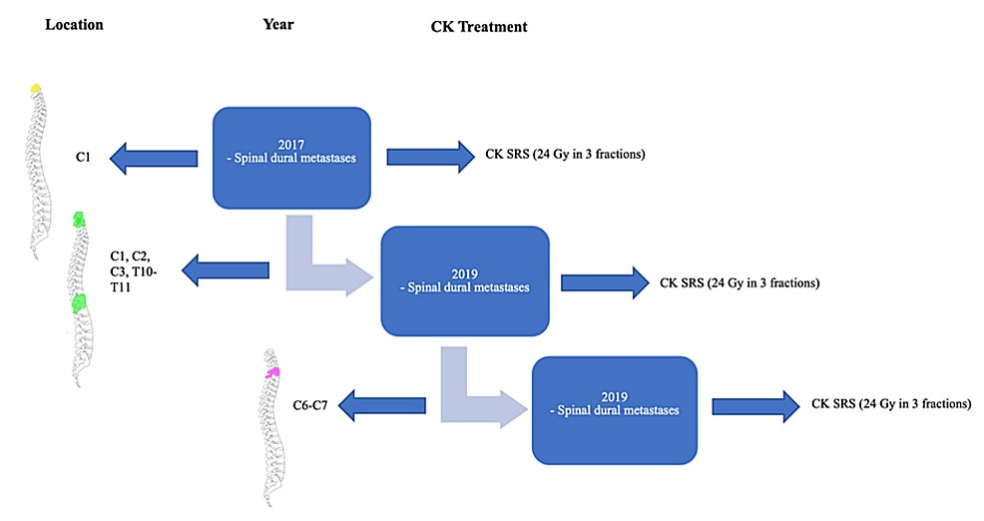
Spinal lesions: CyberKnife treatment details CK SRS: CyberKnife stereotactic radiosurgery

A total of six spinal dural lesions were treated with SRS. The most involved spinal levels were the cervical and thoracic spine. The metastatic lesions appeared first in the cervical spine, which reported good local tumor control (LTC) after treatment with SRS. The lesions remained stable for two years before the appearance of metastases in other locations in the spine, including the cervical spine and thoracic spine. All were treated with SRS. The median target volume was 0.72 cc (range: 0.32-2.54). CK SRS dose was the same for all the lesions. A marginal dose of 24 Gy was delivered in three fractions to the median isodose line of 80% (range: 78-81) to each tumor and a median conformality index of 1.79 (Figure 3). All the lesions remained stable in size at six-, 12-, and 24-month follow-ups. Even though all the lesions were locally controlled at the last follow-up, our patient had multiple new lesions in multiple spinal locations, including, the cervical, thoracic, and lumbar spine, demonstrating progressive disease. The survival observed in our case was 40 months. No adverse radiation effects were reported. Figure 4 depicts the CyberKnife treatment details for spinal lesions.

## Discussion

ENB shows a propensity toward both local and distant recurrences even with aggressive therapy [9]. They spread via direct invasion of the brain parenchyma and metastasize through the lymphatic, hematogenous, or leptomeningeal spread. Of note, 50-60% of patients show local recurrences, while 10-62% of patients report metastatic disease [3]. After invading the cribriform plate, the tumor extends to the anterior skull base and then into the brain parenchyma locally or through the leptomeninges distally [13]. the most common site of metastasis is the cervical lymph nodes. Other locations with reported metastases include the breast, lung, prostate, spine, bone, parotid, viscera, and abdomen [13-15].

Leptomeningeal metastases of ENB are extremely rare. They may be intracranial or spinal and carry a grim prognosis [1]. Less than 20 cases of intracranial leptomeningeal metastases have been reported in the literature so far [15,16,17,18]. Spinal leptomeningeal metastases are even rarer with only six reported cases in the literature [9,12,15,19,20]. It was first reported in 1994 by Louboutin et al., who described meningeal metastases of ENB in the cauda equina. Two more cases were reported in 2002 [19] and one in 2005 [20]. All these patients were treated with chemotherapy and/or radiotherapy.

The treatment for leptomeningeal metastases secondary to ENB typically involves a combination of therapies, including surgery, radiation therapy, and chemotherapy. The specific treatment approach may depend on factors such as the size and location of the metastases, as well as the overall health of the patient. Surgical resection may be considered for localized spinal tumors [9,15], while radiation therapy can be used to treat residual disease or to control symptoms such as pain [20]. Chemotherapy may be used to treat widespread metastasis [12], either as a standalone treatment or in combination with surgery and radiation [19]. The goal of treatment is to control the spread of cancer, reduce symptoms, and improve the overall quality of life for the patient.

Shirzadi et al. [15] and Sivakumar et al. [9] have published case reports with both cranial and spinal leptomeningeal metastases in three patients. None of the previously reported cases of ENB spinal leptomeningeal metastasis was treated with SRS. The survival in these patients ranges from nine months to nine years (Table 2).

**Table 2:** A literature review of spinal leptomeningeal metastases secondary to esthesioneuroblastoma C: cervical; CK: CyberKnife; L: lumbar; SRS: stereotactic radiosurgery; T: thoracic

S. no	Authors	Year	Number of patients	Site of lesion	Treatment	Survival
1	Louboutin et al. [12]	1994	1	Cauda equina (leptomeningeal carcinomatosis)	Intrathecal chemotherapy	9 months
2	Chamberlain [19]	2002	2	T8 subarachnoid nodule; multiple subarachnoid nodules	Chemotherapy and radiotherapy	11 months, 12 months
3	Murakami et al. [20]	2005	1	Subdural C3–C7, L2–L4 (meningeal spread)	Radiotherapy	8 years
4	Shirzadi et al. [15]	2013	1	T8–9, T11	Posterior laminectomy	3 years
5	Sivakumar et al. [9]	2015	1	T8–T10, L3–L4	T8–T10 laminectomies, left T9 costotransversectomy	9 years
6	Present case	2023	1	C1, C2, C3, C6–C7, T10–T11	CK SRS	40 months

We treated six metastatic lesions in our patient with CK SRS. She was monitored for remission or progression via regular MRI scans, and the progression-free survival (PFS), overall survival (OS), and LTC were evaluated. Notably, no radiation-induced adverse events were observed during the course of treatment. Our patient reported a favorable LTC. To the best of our knowledge, this is the first report of the successful treatment of ENB spinal leptomeningeal metastases using SRS.

## Conclusions

Leptomeningeal metastases of ENB to the spine are infrequent, and the scientific literature on this subject is scarce. Treatment with SRS results in relatively favorable long-term survival and local control. There is a need for more extensive studies and clinical trials to provide improved clinical outcome data and ascertain whether SRS is an effective treatment option for ENB leptomeningeal metastases.

## References

[1] Theilgaard SA, Buchwald C, Ingeholm P, Kornum Larsen S, Eriksen JG, Sand Hansen H (2003). Esthesioneuroblastoma: a Danish demographic study of 40 patients registered between 1978 and 2000. Acta Otolaryngol.

[2] Limaiem F, Das JM (2023). Esthesioneuroblastoma. StatPearls Publishing LLC.

[3] Broich G, Pagliari A, Ottaviani F (1997). Esthesioneuroblastoma: a general review of the cases published since the discovery of the tumour in 1924. Anticancer Res.

[4] Levine PA, Gallagher R, Cantrell RW (1999). Esthesioneuroblastoma: reflections of a 21-year experience. Laryngoscope.

[5] Lund VJ, Howard D, Wei W, Spittle M (2003). Olfactory neuroblastoma: past, present, and future?. Laryngoscope.

[6] Bailey BJ, Barton S (1975). Olfactory neuroblastoma. Management and prognosis. Arch Otolaryngol.

[7] Howell MC, Branstetter BF 4th, Snyderman CH (2011). Patterns of regional spread for esthesioneuroblastoma. AJNR Am J Neuroradiol.

[8] Shaari CM, Catalano PJ, Sen C, Post K (1996). Central nervous system metastases from esthesioneuroblastoma. Otolaryngol Head Neck Surg.

[9] Sivakumar W, Oh N, Cutler A, Colman H, Couldwell WT (2015). Cranial and spinal leptomeningeal dissemination in esthesioneuroblastoma: two reports of distant central nervous system metastasis and rationale for treatment. Surg Neurol Int.

[10] Martinez-Perez R, Hardesty DA, Palmer J, Zachariah M, Otto BA, Carrau RL, Prevedello DM (2020). Remote leptomeningeal dissemination in olfactory neuroblastoma mimicking multiple parasagittal meningiomas: diagnostic and therapeutic challenge. World Neurosurg.

[11] Jiang W, Liu J, Gullane PJ, Gentili F, Wharen RE, Kim BY, DeMonte F (2016). Non-contiguous meningeal metastases of olfactory neuroblastoma. J Neurooncol.

[12] Louboutin JP, Maugard-Louboutin C, Fumoleau P (1994). Leptomeningeal infiltration in esthesioneuroblastoma: report of two cases with poor prognosis. Eur Neurol.

[13] Arnold PM, Habib A, Newell K, Anderson KK (2009). Esthesioneuroblastoma metastatic to the thoracic intradural and extradural space. Spine J.

[14] Klepin HD, McMullen KP, Lesser GJ (2005). Esthesioneuroblastoma. Curr Treat Options Oncol.

[15] Shirzadi AS, Drazin DG, Strickland AS, Bannykh SI, Johnson JP (2013). Vertebral column metastases from an esthesioneuroblastoma: chemotherapy, radiation, and resection for recurrence with 15-year followup. Case Rep Surg.

[16] Rodas RA, Erkman-Balis B, Cahill DW (1986). Late intracranial metastasis from esthesioneuroblastoma: case report and review of the literature. Neurosurgery.

[17] Yu T, Xu YK, Li L (2009). Esthesioneuroblastoma methods of intracranial extension: CT and MR imaging findings. Neuroradiology.

[18] Mohindra S, Tripathi M, Mohindra S, Savardekar A, Radotra BD (2015). Multiple intradural spinal metastases of esthesioneuroblastoma: a case report. Br J Neurosurg.

[19] Chamberlain MC (2002). Treatment of intracranial metastatic esthesioneuroblastoma. Cancer.

[20] Murakami M, Kakita K, Kimura S, Hosokawa Y (2005). Subdural extension of recurrent olfactory neuroblastoma. Case report. Neurol Med Chir (Tokyo).

